# Pallidal stimulation for primary generalised dystonia: effect on cognition, mood and quality of life

**DOI:** 10.1007/s00415-013-7161-2

**Published:** 2013-11-01

**Authors:** Marjan Jahanshahi, Mariam Torkamani, Mazda Beigi, Leonora Wilkinson, Donna Page, Laura Madeley, Kailash Bhatia, Marwan Hariz, Ludvic Zrinzo, Patricia Limousin, Diane Ruge, Stephen Tisch

**Affiliations:** 1 Sobell Department of Motor Neuroscience and Movement Disorders, UCL Institute of Neurology, The National Hospital for Neurology and Neurosurgery, Queen Square, London, WC1N 3BG UK; 2 Present Address: Unit of Behavioral Neurology, National Institute of Neurological Disorders and Stroke, Bethesda, MD USA; 3 Present Address: Ballito Medical Center, Ballito, South Africa; 4 Present Address: The Older People’s Liaison Service, Newham University Hospital, East Ham, London; 5 Present Address: Department of Neurology, St. Vincent’s Hospital, Darlinghurst, NSW Australia

**Keywords:** Primary generalised dystonia, Deep brain stimulation, Globus pallidus, Cognition, Executive function

## Abstract

We investigated the effect of pallidal deep brain stimulation (GPi-DBS) in dystonia on cognition, mood, and quality of life and also assessed if DYT1 gene status influenced cognitive outcome following GPi-DBS. Fourteen patients with primary generalized dystonia (PGD) were assessed, measuring their estimated premorbid and current IQ, memory for words and faces, and working memory, language, executive function, and sustained attention, one month before and one year or more after surgery. Changes in mood and behaviour and quality of life were also assessed. There was a significant improvement of dystonia with GPi-DBS (69 % improvement in Burke-Fahn-Marsden score, *p* < 0.0001). Performance on five cognitive tests either improved or declined at post-surgical follow-up. Calculation of a reliable change index suggested that deterioration in sustained attention on the PASAT was the only reliable change (worse after surgery) in cognition with GPi-DBS. DYT1 gene status did not influence cognitive outcome following GPi-DBS. Depression, anxiety and apathy were not significantly altered, and ratings of health status on the EQ5D remained unchanged. In our sample, GPi-DBS was only associated with an isolated deficit on a test of sustained attention, confirming that GPi-DBS in PGD is clinically effective and safe, without adverse effects on the main domains of cognitive function. The dissociation between GPi-DBS improving dystonia, but not having a significant positive impact on the patients’ QoL, warrants further investigation.

## Introduction

Primary generalised dystonia (PGD) is a movement disorder associated with sustained muscle contractions and abnormal postures. Unlike other disorders of the basal ganglia such as Parkinson’s disease (PD), cognitive deficits are not considered part of the clinical presentation or disease progression in PGD. Studies looking at cognitive function in dystonia (summarized in Table 1) have confirmed that patients with primary dystonia do not exhibit major deficits in intellectual ability, attention, memory, or language [1–8]. However, specific deficits on tests of visuo-spatial function relating to egocentric space [9], impaired semantic fluency and dual task performance [2], greater susceptibility to retroactive interference [4], deficits in set-shifting [6], impairment in complex movement planning and motor dexterity, visuospatial working memory and tactile object recognition have been documented in primary dystonia [7]. Some authors [3], but not others [4], have also found a selective deficit in extra-dimensional set-shifting. These differences across studies may be related to the heterogeneity across samples of the exact type and extent of dystonia and/or to the presence/absence of neuropsychiatric symptoms, particularly depression and anxiety, that are common in dystonia and can be associated with cognitive impairment. As previously suggested [2], some of these deficits may be related to the patients’ attempts to control their dystonia during cognitive testing. Supporting this, Allam et al. [5] showed that improvement of blepharospasm following botulinum toxin was associated with significant improvement on a test of sustained attention.

**Table 1:** Studies investigating cognition in primary dystonia using detailed neuropsychological assessment

Author	*N*	Sample characteristics	Neuropsychological battery		Main findings
Taylor et al. [1]	20	All idopathic dystonia with no cognitive impairment?	Wechsler memory scale Tower of Toronto Buschke selective reminding Conditional associative	Learning Stroop Wisconsin Word fluency Block counting	Compared to age matched healthy controls; no differences found prior to medication. However significant difference on *explicit memory* and *reduced speed of information processing* about 2–4 months after taking 15–74 mg of trihexyphenidyl daily
Jahanshahi et al. [2]	10	10 Idiopathic dystonia patients 3 generalized dystonia 5 Cervical dystonia 2 Focal arm dystonia	National adult reading test Word fluency Wisconsin card sorting test Stroop colour word Naming test Missing digit test Self-ordered random number sequence test	Visual conditional associative learning Conditional associative learning tests Paced auditory serial addition test Dual ask performance Repetition random number generation	Only differences between the two groups was on *category word fluency* and *dual task performance*
Scott et al. [3]	14	14 patients with focal, segmental, or generalized dystonia	Cambridge neuropsychological test automated battery Wisconsin card sorting test (set shifting) National adult reading test Raven’s standard progressive matrices The symbol digit modalities test The Stroop test Trail making test Categorical and phonemic verbal fluency	Speed of Comprehension from the speed and capacity of language processing test Recognition memory test Story recall subtest from the adult memory and information processing battery Wechsler adults intelligence scale (digit span) Medical College of Georgia Complex Figures Boston naming test Judgement of line orientation test	*Only deficit on attentional set shifting* on the Cambridge neuropsychological test automated batter. Significant difficulties negotiating the extra-dimensional set-shifting phase of the intra-extra dimensional set shift task.
Balas et al. [4]	28	28 DYT-1 gene carriers with childhood onset generalized dystonia: 20 symptomatic DYT1 dystonia- (SYM) 8 non- symptomatic DYT1 dystonia- (N-SYM)	Rey auditory verbal learning test Rey complex figure Language phonemic verbal fluency, Semantic verbal fluency Trail-making A and B Stroop test Cambridge neuropsychological test automated battery Visual analog scale	Wechsler Adults Intelligence Scale III (digit span and similarities) Symbol search Judgment of line orientation Purdue pegboard Beck depression inventory anxiety Spielberger state and trait anxiety questionnaire	No significant differences between the N-SYM group and the control group on any of the measures. The only significant differences between the SYM group and controls were that the SYM group showed *increased verbal memory retroactive interference*
Allam et al. [5]	9	9 patients with primary carnial dystonia (blepharospasm)	Rey auditory verbal learning test Toulouse-Pieron test Wechsler memory scale-R-digit subtest	Wechsler intelligence scale-R-digit symbol subtest Stroop colour word naming test	There were sustained attentional deficits prior to botox injections. Following botox injections, there were *no significant differences in sustained attention* compared with controls
Bugalho et al. [6]	45	45 patients with primary dystonia (focal and segmental): 15 blepharospasm 15 cervical dystonia 15 writer’s cramp	Wisconsin card sorting test Stroop test Benton’s visual retention test	Block assembly test from Wechsler adults intelligence scale Yale brown obsessive compulsive scale	Patients made *more preservative errors* on *Wisconsin Card sorting test* and had a higher mean *obsessive compulsive* score than controls
Aleman et al. [7]	20	20 patients with primary carnial dystonia (blepharospasm)	Wechsler adults intelligence scale Five digits test-Stroop Raven’s matrices Luria’s task	Purdue pegboard Oral making test Digital recognition Tactile denomination Tapping test Wechsler memory scale	Compared to controls, matched for severity of depression and level of education, the patients showed *impaired*; *complex movement planning*, *motor dexterity*, *visuospatial working memory*, and *tactile object recognition*

Pallidal deep brain stimulation (GPi-DBS) is an established and effective treatment for dystonia [10, 11]. The safety of GPi-DBS for dystonia in relation to cognition has been shown in neuropsychological studies [12, 13] and clinical series following patients for up to three years postoperatively [10, 14], which conclude that GPi-DBS surgery does not affect post-operative cognition in primary dystonia.

The aim of this study was twofold: (1) to investigate the impact of bilateral GPi-DBS in PGD on cognitive function, mood and quality of life, (2) to examine if DYT1 gene status, which has been associated with better post-surgical outcome after GPi-DBS in PGD [15], influences cognition and mood following GPi-DBS.

## Methods

### Sample

Fourteen consecutive patients (10 = female) with PGD undergoing bilateral GPi-DBS at the National Hospital for Neurology and Neurosurgery were included. Demographic and clinical details of the sample are presented in Table 2. The mean age was 41.9 (range 18–64, SD = 18.5) and mean duration of illness was 24.1 years (SD = 16.7). Seven patients tested positive for the DYT1 torsion gene. The Burke-Fahn-Marsden Scale [16] was used to assess the severity of dystonia.

**Table 2:** Demographic and clinical details of the dystonia patients

	Gender	Age (years)	Disease duration (years)	DYT 1 gene status	BFM score pre-op (0–120)	BFM score post-op (0–120)	Medication	DBS parameters
1	F	18	9	+	48	0	Trihexyphenidyl	R: 4,5-, 3.9v, 60:μs, 130 Hz L: 0,1-, 3.9v, 60:μs, 130 Hz
2	M	24	16	−	73	16	Clonazepam	R: 4,5-, 3.9 v, 90:μs, 130 Hz L: 0,1-, 3.8 v, 90:μs, 130 Hz
3	M	47	37	+	52	24	Diazepam tetrabenazine	R: 4-, 3.5v, 90:μs, 130 Hz L: 0,1-, 3.3v, 60:μs, 130 Hz
4	F	23	12	−	53	33	None	R: 5-, 4.0v, 60:μs, 130 Hz L: 1-, 4.2v, 60:μs, 130 Hz
5	F	52	36	+	29	9	None	R: 4-, 3.0v, 60:μs, 130 Hz L: 0-, 3.0v, 60:μs, 130 Hz
6	F	16	7	+	20	4	l-Dopa trihexyphenidyl	R: 4-, 2.9v, 60:μs, 130 Hz L: 0-, 2.9v, 60:μs, 130 Hz
7	F	63	4	−	23	1	l-Dopa trihexyphenidyl	R: 6-, 4.6v, 90:μs, 130 Hz L: 2-, 4.6v, 90:μs, 130 Hz
8	F	54	43	−	24	12	None	R: 4-, 3.7v, 60:μs, 130 Hz L: 1-, 3.7v, 60:μs, 130 Hz
9	F	36	25	+	21	1	Trihexyphenidyl	R: 4-, 4.6v, 90:μs, 130 Hz L: 0-, 4.2v, 90:μs, 130 Hz
10	M	62	43	−	28	15	Trihexyphenidyl Baclofen clonazepam	R: 6-, 3.7v, 90:μs, 130 Hz L: 1-, 3.7v, 90:μs, 130 Hz
11	F	22	15	+	51	11	Trihexyphenidyl Baclofen	R: 4-, 3.6v, 60:μs, 130 Hz L: 0-, 3.6v, 60:μs, 130 Hz
12	F	64	50	−	46	17	None	R: 5,6-, 3.5v, 60:μs, 130 Hz L: 1,2-, 3.5v, 60:μs, 130 Hz
13	M	62	7	−	64	38	None	R: 4,5-, 3.5v, 60:μs, 130 Hz L: 1,2-, 3.5v, 60:μs, 130 Hz
14	F	42	30	+	27	7	None	R: 5-, 3.5v, 60:μs, 130 Hz L: 1, 3.5v, 60:μs, 130 Hz

### Neuropsychological assessment

Patients were assessed prior to surgery, and one year or more post-operatively (mean duration of follow-up 14.4 ± 4.5 months).

The assessment battery included the following tests, which were administered according to standard procedures (for details of tests please see [2, 17]).

The Mini Mental State Examination (MMSE) [18] for global cognitive functioning, the National Art Reading Test (NART) [19] for premorbid IQ and the Wechsler Adult Intelligence Test-Revised (WAIS-R) [20] for current verbal IQ were used. The Auditory Verbal Learning Test (RAVLT) [21], the recognition memory for faces-short form (RMF) [22] ^,^ and the Self-Ordered Pointing Test [23] were respectively used to assess episodic memory for words, memory for non-verbal material and working memory. The phonemic, semantic and alternating versions of the Verbal Fluency Test [24] and the Graded Naming Test [25] were employed for language assessment. Tests of executive function included the Modified Wisconsin Card Sorting Test (MWCST) [26] for maintaining and shifting ‘set’, the Stroop colour word naming task [27] to measure inhibition of proponent/habitual responses, and the Trial Making Test [28] for behavioural regulation and task switching. The Paced Auditory Serial Addition Test (PASAT) [29] was used to assess sustained attention.

The Beck Depression Inventory (BDI) [30], Beck Anxiety Inventory (BAI) [31], and the Apathy Evaluation Scale (AES) [32] were used to measure depression, anxiety and apathy, respectively. Higher scores on these measures are indicative of high depression, anxiety and apathy. The EQ5D [33], which covers five quality of life (QoL) dimensions (mobility, self-care, usual activities, pain/discomfort and anxiety/depression), was used to measure QoL. The EQ5D includes a visual analogue scale (VAS) used for rating ‘current health state’ with a range of 0(worst imaginable health)–100(best imaginable health), and the five subscores of the test can be combined to give a summary index value of 0–1, with lower scores representing poorer overall QoL.

The study was approved by the Joint Research Committee of the National Hospital for Neurology and Neurosurgery and the Institute of Neurology, London, UK. All patients gave written informed consent.

## Results

### Pre vs. post-operative change in dystonia

Mean BFM score was 39.9 (SD = 17.4) at baseline and 13.3 (SD = 11.6) after surgery. Dystonia was significantly improved from before to after surgery[*t*(13) = 7.6, *p* < 0.0001]. The average improvement in dystonia was 68.7 % (±20.8).

### Pre vs. post-operative change in cognitive function

At baseline, the mean predicted premorbid IQ from the NART was 104.5 (SD = 13.2), which is in the average range. With *p* < 0.05, there was a significant change in scores for five cognitive measures (see Table 3). Three of these indicated poorer performance after surgery, and two were suggestive of post-operative improvement. The scaled score on digit span was lower[*t*(11) = 2.6, *p* = 0.024], fewer items were recalled on RAVLT (Trial 5)[*t*(13) = 2.6, *p* = 0.022], and there was a significant increase in errors on PASAT[*t*(8) = −2.9, *p* = 0.021]. In contrast, accuracy on the Stroop control task improved [*Z* = 2.2, *p* = 0.027] and there were fewer non-perseverative errors on the MWCST [*Z* = 2.5, *p* = 0.013] after surgery. Inspection of the individual data showed an average reduction of three points on the WAIS-R digit span and two points on the RAVLT (range +2 to −7 on both tests), and an increase of eight errors on the PASAT at follow-up compared to baseline.

**Table 3:** Mean and standard deviations (SD) of the pre-surgical and post-surgical scores for the dystonia patients on the measures of cognitive function

Measure	Pre-surgery mean (SD)	Post-surgery mean (SD)	*p* value
Mini mental state examination (max = 30)	27.20 (2.90)	27.60 (2.32)	0.343
WAIS-R subtests			
Digit span	10.42 (3.31)	7.92 (3.89)	0.024*
Similarities	12.33 (3.55)	10.83 (3.24)	0.124
Vocabulary	9.92 (3.55)	10.17 (3.46)	0.699
Verbal IQ (prorated)	103.73 (14.02)	93.36 (11.96)	0.094
RAVLT			
Correct recall: Trial 1 (max 15)	5.79 (2.36)	5.71 (3.02)	0.932
Correct recall: Trial 5 (max 15)	12.64 (2.02)	11.00 (3.61)	0.022*
Recall intrusions	0.79 (0.97)	0.93 (1.33)	0.699
Recall repetitions	0.93 (1.21)	0.64 (1.15)	0.470
Recognition: total correct (max 15)	14.36 (1.27)	13.43 (2.31)	0.084
Recognition: false positives	0.86 (1.09)	1.36 (2.37)	0.390
Short recognition memory for faces			
Total correct (max 25)	23.86 (1.41)	24.07 (2.20)	0.664
Subject-ordered pointing			
4 items (mean errors)	0.10 (0.32)	0.40 (0.52)	0.081
8 items (mean errors)	1.60 (0.97)	1.10 (0.88)	0.244
12 items (mean errors)	2.10 (1.60)	2.20 (1.03)	0.853
Verbal fluency total words generated			
Letter fluency	41.85 (18.49)	39.85 (15.89)	0.457
Category fluency	15.23 (5.49)	16.00 (6.05)	0.409
Category switching fluency	18.49 (5.13)	15.89 (4.40)	0.673
Graded naming test			
Total correct (max 30)	19.29 (4.84)	20.07 (5.56)	0.085
Modified Wisconsin card sorting			
Categories correctly sorted (max = 6)	5.73 (1.27)	5.73 (0.65)	1.00
Total errors	7.17 (5.22)	4.75 (3.77)	0.115
Perseverative errors	1.58 (1.62)	2.08 (3.03)	0.568
Non-perseverative errors	5.50 (4.36)	1.92 (1.98)	0.016*
Stroop			
Control colour naming time(ms)	76.43 (21.54)	81.1 (25.12)	0.436
Control colour naming number of errors	1.38 (1.71)	0.38 (0.65)	0.02*
Stroop interference time(ms)	126.47 (44.61)	144.21 (50.59)	0.107
Stroop interference numbers of errors	2.61 (4.03))	1.84 (4.91)	0.471
Difference score in completion time (ms)	50.04 (30.52)	63.14 (31.98)	0.168
Trails A and B			
Trails A: completion time (ms)	57.24 (33.43)	61.61 (36.76)	0.409
Trails B: completion time (ms)	115.17 (78.26)	116.38 (66.6)	0.952
Difference Trail B−A (ms)	57.93 (65.87)	54.76 (48.25)	0.886
PASAT			
Total errors (max 30)	6.55 (8.39)	14.56 (13.45)	0.02*

*p < 0.05

To determine if the significantly lower performance at follow-up was statistically reliable, we calculated the reliable change index (RCI) for the related measures (Table 4). RCI determines if change in a score from before to after an intervention is statistically reliable by taking into account the reliability of the measure [34]. The formula for calculating it is: ${\text{RCI}} = x_{ 2} - x_{ 1} /S_{\text{diff}} \left( {{\text{baseline score}} - {\text{follow - up score}}/{\text{the standard error of difference}}} \right).$ The *S*_diff_ is calculated as $\surd 2(S_{\text{E}} )$, with *S*_E_ being the standard error of measurement. The results are then grouped into three categories showing the percentage of: reliable improvement, reliable decline, or no change. The greatest average change was observed for PASAT, and the RCI echoes this, showing that at follow-up more participants showed deterioration (45 %) than improvement (33 %) or no change (22 %). Although only one patient made fewer errors on the PASAT at follow-up compared to baseline (one less error), the 33 % improvement calculated by the RCI takes into account practice effects and is based on this patient and two others; one with only one additional error and the other with no additional errors. In contrast, RCI for WAIS-R digit span showed no change in 58 %, improvement in 25 % and deterioration in only 17 % post-surgery. Similarly, the RCI result for RAVLT (Trial 5) indicated no change in 50 % of participants, and although 21 % had declined, 29 % had improved. Thus, the RCI results suggest that only the change in PASAT was clinically significant.

**Table 4:** Reliable change indices for the three cognitive measures showing post-operative decline in performance

Measure	% Declined	% No change	% Improved	95 % RCI criterion
WAIS-R subtests				
Digit span	16.67 %	58.33 %	25.00 %	2.10
RAVLT				
Words recalled on Trial 5	21.43 %	50.00 %	28.57 %	1.30
PASAT				
Errors	44.44 %	22.22 %	33.33 %	5.20

*WAIS-R* Wechsler adult intelligence scale, *RAVLT* Rey adult verbal learning test, *PASAT* Paced auditory serial addition test

### Pre-operative vs. post-operative change in mood

There were no significant changes in depression or anxiety from baseline to follow-up [BAI: *t*(7) = −0.201, *p* = 0.846; BDI: *t*(8) = −0.174, *p* = 0.866]. Based on the scale’s cut-off points, on average the patients had mild anxiety and no depression, both before and after surgery (see Table 5). Similarly, there was no significant change in apathy [AES: *t*(8) = −0.809, *p* = 0.442], or apathy subscores (all *p* > 0.05).

**Table 5:** Self-ratings of depression, anxiety, apathy, effort, physical and mental fatigue, and quality of life by the patients with dystonia before and after surgery

Measure (range)	Baseline	Follow-up	*p* value
Beck Depression Inventory (0–63)	7.22 (4.27)	7.44 (6.42)	0.866
Beck Anxiety Inventory (0–63)	11.89 (5.94)	12.38 (10.34)	0.846
AES-total apathy score (18–72)	26.89 (3.79)	27.78 (4.09)	0.442
AES: emotional subtest (2–8)	3.78 (1.20)	3.89 (1.45)	0.594
AES: cognitive subtest (8–32)	11.67 (2.29)	12.22 (2.39)	0.516
AES: behavioural subtest (5–20)	7.11 (1.36)	7.22 (1.39)	0.834
AES: other items (3–12)	4.33 (1.50)	4.44 (1.33)	0.842
Effort (0–10)	5.81 (3.54)	6 (2.76)	0.779
Physical fatigue (0–10)	5.00 (3.00)	4.09 (2.66)	0.138
Mental fatigue (0–10)	5.73 (3.64)	5.64 (3.44)	0.911
EQ5D domain			
Mobility	92.31 %	75.00 %	0.167
Self care	30.77 %	25.00 %	1.00
Usual activities	61.54 %	50.00 %	0.167
Pain/discomfort	84.62 %	58.33 %	0.082
Anxiety/depression	30.77 %	50.00 %	0.167
EQ5D summary index	0.61 (0.22)	0.73 (0.15)	0.141
EQ5D health statue (VAS)	68.45 (20.47)	69.27 (17.66)	0.921

*AES* Apathy evaluation scale

### Pre-operative vs. post-operative change in quality of life

There was no significant difference between assessments in the EQ5D summary index [*t*(10) = 1.589, *p* = 0.141] or VAS[*t*(10) = -0.101, *p* = 0.921]. However, the percentage of patients experiencing problems was reduced post-surgery compared to baseline in all EQ5D domains except anxiety/depression, although these differences were not significantly different (see Table 5). Correlation analyses showed no statistical correlation between change in dystonia and change in QoL from before to after surgery (EQ5D summary index: *r* = −0.618, *p* = 0.057; EQ5D vas, *r* = −0.115, *p* = 0.751).

### DYT1 gene status and cognitive outcome with GPi-DBS

A series of two-way ANOVAs with Group (DYT1 positive, vs. negative) as the between-groups variable and time of assessment (pre-surgery vs. post-surgery) as the within-subject variable were completed. The results revealed only a main effect of time for WAIS-R digit span [*F*(1,10) = 6.66, *p* < 0.05], RAVLT (Trial 5) [*F*(1,12) = 6.25, *p* < 0.05] and PASAT [*F*(1,7) = 8.84, *p* < 0.05] (see Table 3), but no significant interactions. Therefore, the DYT1 gene status did not influence cognitive outcome following GPi-DBS.

### Correlation of demographic and clinical features with change in cognitive scores with GPi-DBS

We examined the association of clinical and demographic features and change in cognitive measures using Pearson correlation coefficients. Duration of illness correlated with changes in trail making (version B) [*r*(14) = −0.58, *p* < 0.03] and trail making difference score (version B−A) [*r*(14) = −0.60, *p* < 0.02]. Age of onset correlated with changes in trail making (version B) [*r*(14) = 0.55, *p* < 0.04]. The BMF pre-operative scores correlated with changes in WAIS-R (verbal IQ) [*r*(14) = 0.61, *p* < 0.02], category fluency [*r*(13) = 0.615, *p* < 0.03] and switching [*r*(13) = 0.57, *p* < 0.05].

The pulse width of the right contact correlated with change in WAIS similarities [*r*(14) = 0.55, *p* < 0.04]. The amplitude of DBS on the right and left contact correlated with changes in RAVLT (Trial 5) [*r*(14) = 0.68, *p* < 0.01 and *r*(14) = 0.59, *p* < 0.03, respectively]. There were no other significant correlations, including between the stimulation parameters and change in PASAT scores (all *p* > 0.05). Furthermore, we did not find a significant difference in PASAT change scores between patients with high (90 μs) versus low (60 μs) pulse width (*p* > 0.05). Similarly with a median split on the DBS amplitudes, patients with high versus low stimulation amplitudes did not show any significant differences in PASAT change scores (*p* > 0.05).

## Discussion

The results of this study confirmed the benefits of GPi-DBS for PGD, showing a significant post-surgical improvement. GPi-DBS did not affect IQ, memory, language and executive function. The only reliable change following surgery was worse performance on PASAT. DYT1 gene status did not influence post-surgical cognitive outcome. There were no significant changes in depression, anxiety or apathy after surgery. Despite fewer patients experiencing problems in four of the EQ5D domains after surgery, the differences were not significant.

In line with previous literature, our results show no change in most aspects of cognition following GPi-DBS or subthalamic(STN)-DBS, and show variability in cognitive outcomes, with findings of deterioration, improvement, and no change [10, 14, 35]. In a case series, Kleiner-Fisman et al. [35] reported a mild but non-significant decline in executive function, and significantly worse verbal and visual memory after STN-DBS. Similarly, Kiss [14] reported significant decline in verbal fluency in one case and in verbal memory in another but without any impact on the patient’s daily life. In our study, RCI calculations showed that after GPi-DBS only the deterioration in the PASAT was clinically significant; whereas deterioration in digit span and RAVLT(Trial 5), and improvement on MWCST and stroop (control task) were not reliable.

Close inspection of the PASAT data showed that in all but one participant, error scores had increased at follow-up. PASAT requires participants to hold the last number presented ‘on line’ in working memory, add it to the next number, and provide their sum. Errors are often linked to failure to respond, due to the inability to maintain attention/information in working memory, and/or to manipulate the information to generate a response. GPi-DBS could have interfered with any of these processes, resulting in poorer performance. The change in PASAT scores did not have any significant associations with the DBS parameters. The point of entry of the electrodes is likely to be through the supplementary motor area (SMA, BA 6). However, any ‘insult’ to frontal areas during electrode entry is minimal, and it is likely that any effects relate to chronic stimulation of the motor front-striatal circuit. In the motor circuit there are separate projections from the GPi to the motor cortex, SMA and the lateral part of Brodmann area six, the lateral premotor cortex [36]. Furthermore, the SMA and the lateral premotor cortex are hyperactive in primary dystonia [37]. The SMA and the lateral premotor cortex are among the areas activated during performance of the PASAT [38], and have been shown in animal experimentation to be involved in response selection on the basis of internal versus external cues [39], processes that are involved in the PASAT in addition to working memory. It is thus possible that the impaired performance on the PASAT following GPi DBS in the present sample relates to the effects of GPi stimulation on these medial and lateral premotor areas. This proposal needs to be tested in future studies.

Some studies have reported improvements in performance on tests of cognitive function after GPi-DBS for children with secondary dystonia [40] and adults with dystonia [13]. Pillon et al. [13] reported mild but significant improvement in performance on Raven progressive matrices, WAIS-R (similarities), MWCST (non-perservative errors) and free verbal recall, which were all maintained at three years follow-up [10]. Halbig et al. [12] also found a significant post-surgical improvement on the trail making test (version A) after surgery. However, the pre-operative assessment of cognition may have been affected by the severity of dystonia and thus the post-operative improvement may partly reflect the symptomatic improvement. Where the interval between baseline and follow-up have been short, practice effects may have influenced performance. Also, the effects of anti-cholinergic medication used for the treatment of dystonia on cognition and changes of this medication following GPi-DBS needs to be considered. In this sample, there were no changes in medication between baseline and the post-surgical assessment. To avoid the confounding effects of medication changes on the time course of clinical improvement in dystonia after GPi-DBS, no changes were made in medication for the first 12 months, so the 12 month post-op medications remained the same as baseline. However after 30 months, four of the six patients on Artane were able to reduce their dose by a mean of 36–11 mg, and four others stopped or reduced sulpiride, l-Dopa, baclofen, and/or diazepam by 24 months.

Patients positive for the DYT1 gene are shown to benefit more from GPi-DBS in some [15] but not all studies [10, 11]. Our results show that DYT1 gene status did not influence change in cognition with GPi-DBS. However, correlational analyses suggest association between key clinical variables and cognitive change following GPi-DBS. A shorter duration of illness, older age of onset and more severe dystonia prior to surgery were associated with greater change in executive function and verbal IQ with GPi-DBS. Higher amplitude and pulse width of GPi-DBS were associated with greater change in concept formation and verbal memory.

Despite significant improvement of dystonia, depression and anxiety remained unchanged in our sample. The lack of significant change in mood following GPi-DBS may reflect the mild anxiety and absence of depression at baseline. The majority of previous studies excluded cases with major psychiatric illness from surgery [8]. While some have found that mild-moderate depression improves after GPi-DBS in dystonia [12, 14]; others have not [10, 11]. Generally, anxiety scores do not change with GPi-DBS[8]. These suggest that depression and anxiety can remain unchanged following successful GPi-DBS in dystonia, as found in the current study. Suicide has been documented in association with DBS in dystonia. Foncke et al. [41] reported that two of their 16 participants treated with GPi-DBS committed suicide. Suicide is preventable, and regular monitoring of operated patients, including direct enquiry about suicidal ideations is highly recommended [8].

QoL is impaired in dystonia and a greater percent of dystonia patients report problems on all EQ5D domains compared to the general population [42]. After GPi-DBS, a lower percent of patients in this study reported problems on all EQ5D items except depression/anxiety. However, the summary index and VAS were not significantly altered by surgery, and correlation analysis revealed no significance between improvement in dystonia and change in QoL. Although some studies report post-surgical improvement in most QoL domains in dystonia [43], others suggest that surgery primarily improves the physical domains and to a lesser extent the emotional components of QoL [10]. Given that surgery aims to improve QoL, a lack of positive impact of improvement in dystonia on QoL is at first sight puzzling and of clinical concern. Why doesn’t the significant improvement of dystonia translate into significant improvement of QoL? One possibility is that generic measures may not be sensitive in picking up changes unique to PGD. The EQ5D measures mainly the physical parameters of health, and the lack of improvement in QoL may reflect the simplicity of the measure. Alternatively, our current assessment techniques may not be targeting key patient concerns, highlighting the need to also assess their expectations. Also, while GPi-DBS produces relatively rapid improvement of dystonia, psychosocial adjustment to the functional improvements may require longer time. We have some evidence for this from interviews with operated patients [44], who expressed a need to adapt to their ‘new and improved’ body. This was eloquently expressed by one patient “Now, even though I have been given a new body, I haven’t been given a new mind. It’s like plastic surgery, you might change your nose but how you feel about yourself is still the same”. Such dissociation between the physical change and psychological adjustment may be quantifiable with longitudinal assessments of changes in dystonia symptoms, mood and QoL, thus warranting further investigation.

In conclusion, our results confirm that GPi-DBS is an effective treatment for PGD without producing any major adverse impact on cognition, other than isolated deterioration of performance on a test of sustained attention. Despite significant improvement of dystonia with GPi-DBS, surgery did not significantly alter depression, anxiety, apathy or QoL. This dissociation between GPi-DBS improving dystonia but not having a significant positive impact on the patients’ QoL warrants further investigation.
